# Effects of Intranasal Pseudorabies Virus AH02LA Infection on Microbial Community and Immune Status in the Ileum and Colon of Piglets

**DOI:** 10.3390/v11060518

**Published:** 2019-06-05

**Authors:** Chuanjian Zhang, Yamei Liu, Saisai Chen, Yongfeng Qiao, Yating Zheng, Mengwei Xu, Zhisheng Wang, Jibo Hou, Jichun Wang, Hongjie Fan

**Affiliations:** 1 Institute of Veterinary Immunology and Engineering, National Research Center of Engineering and Technology for Veterinary Biologicals, Jiangsu Key Laboratory for Food Quality and Safety-State Key Laboratory Cultivation Base of the Ministry of Science and Technology, Jiangsu Academy of Agricultural Sciences, Nanjing 210014, China; zcj6717855@126.com (C.Z.); lym613@hotmail.com (Y.L.); saisaichen1990@126.com (S.C.); qiaoyf1990@163.com (Y.Q.); yt851110@163.com (Y.Z.); xumengwei1746109@aliyun.com (M.X.); zhisheng.wang@163.com (Z.W.); houjiboccvv@163.com (J.H.); 2 Jiangsu Co-Innovation Center for Prevention and Control of Important Animal Infectious Diseases and Zoonoses, Yangzhou 225009, China; 3 College of Veterinary Medicine, Nanjing Agricultural University, Nanjing 210095, China

**Keywords:** intranasal PRV AH02LA infection, microbial community, microbial metabolites, immune status, piglets

## Abstract

Pseudorabies virus (PRV) variants broke out in china since 2011, causing high fever, respiratory distress, systemic neurological symptoms, and diarrhea in piglets. This study investigated the effect of intranasal PRV variant (AH02LA) infection on ileal and colonic bacterial communities and immune status in piglets. Ten piglets (free of PRV) were assigned to PRV variant and control groups (uninfected). At day 5 after inoculation, all piglets were euthanized. No PRV was detected in the ileal and colonic mucosa. In the PRV group, we observed up-regulation of specific cytokines gene expression, down-regulation of intestinal barrier-related gene expression, and reduction of secretory immunoglobulin A (sIgA) concentration in the ileum and colon. PRV infection increased the diversity of ileal bacterial community composition. PRV infection reduced the abundance of some beneficial bacteria (*Lactobacillus* species in the ileum and colon; butyrate-producing bacteria species in the colon) and increased the abundance of potentially pathogenic *Fusobacterium nucleatum* in the ileum and *Sphingomonas paucimobilis* in the colon. Moreover, PRV infection decreased concentrations of the beneficial lactate in the ileum and butyrate in the colon. However, this study does not allow to evaluate whether the observed changes are directly due to the PRV infection or rather to indirect effects (fever, clinical signs and changes in diet), and will be our next research content. In summary, our findings provide evidence that intranasal PRV infection directly or indirectly brings gut health risks and implications, although no PRV was detected in the ileum and colon.

## 1. Introduction

Pseudorabies virus (PRV) is a member of the family *Herpesviridae*, subfamily *Alphaherpesvirinae* and can infect most mammals including pigs, cattle, sheep, and dogs [1]. Pseudorabies outbreaks caused by new emerging PRV variants have occurred among the widely used PRV Bartha-K61-vaccinated pigs since 2011, leading to 50% mortality rates in piglets, and 10–30% mortality in growing and finishing pigs [2]. Airborne transmission is believed to be the main mode of PRV transmission. Infected pigs, particularly weaning and starter piglets showed various clinical symptoms, including high fever, respiratory distress, systemic neurological symptoms, and diarrhea [3,4,5]. Previous studies have shown that respiratory and neurological symptoms induced by intranasal PRV infection are related to extensive viral replication and immune response in the respiratory tract and the central nervous system of piglets [6,7]. However, it is not known whether direct infection of the intestine with PRV leads to diarrhea. As a front-line defence, the intestinal immune system can tolerate gut commensal microbiota, and resist pathogens and virus infection [8]. The effects of intranasal PRV infection on both immune status and viral replication in the intestine have not been characterized.

Commensal microbiota plays an important role for the health and development of animals as they stimulate the immune system, resist pathogens and virus, and improve energy harvest [9,10]. Moreover, microbial metabolites influence gut homeostasis. Short-chain fatty acids (SCFAs) are mainly made from carbohydrates and are beneficial to the intestinal health. Lactate is predominant in the small intestine, and can decrease intestinal pH to prevent proliferation of pathogenic bacteria [11]. Previous studies have linked shifts in microbial community dysbiosis to host disease [12,13]. When conditions in the host are unfavorable, such as viral infection, an altered intestinal tract environment may lead to the imbalance of intestinal bacteria that induces or aggravates intestinal inflammation. Most studies have focused on the effect of viral gastrointestinal infections on the gut microbiota [14,15]. However, limited information exists about the effect of non-gastrointestinal infection on gut microbiota. Previous studies have shown that intranasal PRV infection causes diarrhea and necrotizing enteritis in weaning and starter piglets [2,16], suggesting that PRV may affect gut health in piglets. It is unclear whether these symptoms are related to alterations in the intestinal microbiota in infected pigs. Therefore, studies on the intestinal bacterial community in response to intranasal PRV infection are therefore urgently required.

It is well known that different intestinal regions are distinguished from each other in the morphology and function. In the ileum, peyer’s patches are especially predominant, which are essential for maintenance of microbiota accommodating immune homeostasis via dedicated luminal antigen-sampling [17]. The colonic immune system involving in the production of a thick mucus layer, the generation of IgA antibodies, and the presence of large numbers of regulatory T cells, keeps gut microbiota at bay and prevents inflammatory responses against them [17]. Compared to the duodenum and jejunum, the ileum and colon (espiecally colon) have higher numbers of microbiota, which are equipped with stronger fermentative capability to generate various microbial metabolites [18]. This enriched microbiota and microbial fermentation products are more important to gut health and host metabolism [19]. Therefore, the ileum and colon were selected to investigate the effect of PRV on the microbial communities and immune status in the intestine of piglets.

In 2012, we isolated a PRV variant AH02LA from the brain of a dead 1-day-old piglet in a pig farm of Anhui province in China [20,21]. Sequence analysis reveals that AH02LA belongs to the same clade as the other new PRV variants isolated after 2011 in China. Intranasal PRV AH02LA infection caused 100% morbidity or mortality in both 28- and 70-day-old pigs, and major clinical symptoms in weaned pigs are similar to that of other virulent strains and classical strains including high fever, respiratory distress, depression, anorexia, systemic neurological symptoms, and diarrhea. In this study, we aimed to explore whether PRV spread to the intestine after a primary intranasal infection, and investigate the responses of intestinal microbial community and immunity to AH02LA infection. Our results suggest that PRV AH02LA strain negatively affected gut homeostasis at day 5 post intranasal infection, although no PRV propagation was detected in the ileal and colonic mucosa. These findings provide insights into our understanding of the impact of intranasal PRV infection on gut health.

## 2. Materials and Methods 

The experiment was approved by the Institutional Animal Care and Use Committee at the Jiangsu Academy of Agriculture Sciences (Permit number: SYXK (Su) 2015-0019, Approval date: 25 June 2015) and performed in strict accordance with the guidelines provided by the Institutional Biosafety Committee.

### 2.1. Experiment Design and Sample Collection

Ten 28-day piglets free of PRV, porcine reproductive and respiratory syndrome virus (PRRSV), porcine parvovirus (PPV), porcine circovirus 2 (PCV2) and classical swine fever virus (CSFV) were randomly assigned to one of the two groups based on equal body weights. PRV, PRRSV, PPV, PCV2, and CSFV were detected using serological methods and PCR. Enzyme-linked immunosorbent assay was used to detect against PRV (IDEXX, Westbrook, ME, USA), PRRSV (IDEXX, Westbrook, ME, USA), PCV2 (Jeno Biotech, Seoul, Korea), and CSFV (IDEXX, Westbrook, ME, USA) specific antibodies accordance with the manufacturer’s instructions. Antibody titers against porcine parvovirus (PPV) were determined by a haemagglutination inhibition test as described previously [22]. Moreover, PCR or reverse transcription PCR were performed to detect of a panel of potential pathogens including PRV, PRRSV, PPV, PCV2, and CSFV following published procedures [23,24]. The two groups were housed in separated facilities. Piglets in group 1 were inoculated intranasally with 2LD_50_ PRV AH02LA per piglets. Piglets in group 2 were inoculated intranasally with equal volume of Dulbecco’s modified Eagles medium (DMEM). A previous study showed that high-level PRV DNA concentration was detected in the nasal mucosa, lung, spleen, liver, and brain of 2-week-old piglets at day 4 post infection and 15-week-old pigs at day 5 post infection, and extensive viral replication associated with a robust expression of cytokine mRNA was detected in the brain of pigs [6]. Therefore, at day 5 after inoculation, all pigs were euthanized by intravenous injection of pentobarbital sodium (100 mg/kg). During the 5-day experiment, the piglets were fed twice daily (at 8:00 and 17:00, equal-but-adequate food of each meal). Water were consumed *ad libitum*. Body temperature and clinical signs were monitored and recorded daily. Brain and lung samples were collected and immediately frozen at −70 °C until analysis. Mucosa scrapings from the middle section of the ileum and colon were collected using a sterile glass slide and frozen stored at −70 °C for RNA isolation and DNA extraction. In addition, digesta of ileum and colon were collected and immediately frozen at −70 °C until further DNA extraction, metabolite analysis and secretory immunoglobulin A (sIgA) determination.

### 2.2. Pseudorabies Virus (PRV) DNA Detection in the Ileal and Colonic Mucosa

Genomic DNA was extracted from the ileal and colonic mucosa, brain, and lung using a QIAamp DNA kit (Qiagen, Valencia, CA, USA) according to the manufacturer’s instructions. A pair of primers (*gE* part F and *gE* part R; Table S1) were used to detect PRV DNA. Viral load in the ileal and colonic mucosa was quantified by real-time quantitative PCR using specific primers (*gE*-Sybr-F and *gE*-Sybr-R; Table S1) and SYBR Green Premix (Takara Biotechnology, Dalian, China) in Roche Light Cycler^®^ 480 system (Roche Diagnostics, Burgess Hill, UK). Quantification of PRV copy numbers in each sample was performed in triplicate. PRV copy numbers in the ileum or colon were quantified according to standard curves which were generated from serial dilutions of target gene fragment cloned into a pMD-19T vector (TaKaRa Biotechnology, Dalian, China).

### 2.3. Gene Expression Analysis in Ileal and Colonic Mucosa

Total RNA was isolated from ileal and colonic mucosa using TRIzol reagent [25]. Reverse transcription reactions were performed using a PrimeScript® RT Reagent Kit with gDNA Eraser (Takara Bio) according to the manufacturer’s instructions. Real-time quantitative PCR assay for immune-related genes (interleukin-1β (*IL-1β*), *IL-6*, *IL-8*, *IL-10*, interferon-γ (*IFN-γ*), tumor necrosis factor-α (*TNF-α*), *Mucin-1*, *Mucin-2*, *Occludin*, and zonula occludens-1 (*ZO-1*)) in the ileal and colonic mucosa were performed using SYBR Green PCR reagents on Roche Light Cycler^®^ 480 system (Roche Diagnostics, Burgess Hill, UK). The PCR mixture contained 2.0 μL of complementary DNA template, 10 μL of 2 × SYBR Green PCR Master Mix, 0.4 μL of each primer, 0.4 μL of ROX Reference Dye and PCR-grade sterile water to a final volume of 20 μL. The program for PCR amplification was performed using the following conditions: 95 °C for 30 s, followed by 40 cycles composed of 5 s at 95 °C and 30 s at 60 °C, and one cycle of 15 s at 95 °C, 60 s at 60 °C, and 15 s at 95 °C. The primers for immune-related genes were listed in Table S1. Each cDNA was analyzed in triplicate, and gene expression levels of each sample were normalized relative to *β-actin*, *GAPDH*, and *18S rRNA* using the 2^-^^ΔΔ^^Ct^ method. Similar results were obtained and only showed the results using *β-actin* as a normalizer.

### 2.4. sIgA Concentration Analyses in the Ileal and Colonic Digesta

Ileal and colonic digesta (0.5 g) were mixed with 4.5 mL of physiological saline. sIgA concentration in each sample was determined using ELISA kits (Beijing 4A Biotech Co., Ltd., Beijing, China) according to the manufacturer’s instructions.

### 2.5. DNA Extraction, MiSeq Sequencing and Bioinformatics Analyses

Total genomic DNA in the digesta from ileum and colon were extracted with a bead-beating and phenol-chloroform extraction [26]. The V3–V4 regions of the bacterial 16S rRNA genes were amplified by PCR using a universal primer 338F (5′-ACT CCT RCG GGA GGC AGC AG-3′) and 806R (5′-GGA CTA CCV GGG TAT CTA AT-3′) [27]. PCR products were purified with the AxyPrep DNA Gel Extraction Kit (Axygen Biosciences, Union City, CA, USA) following the manufacturer’s instructions. Purified amplicons were pooled and paired-end sequenced (2 × 250) was performed on an Illumina MiSeq platform following the standard protocols.

As previously described [28], the sequences from the Illumina MiSeq platform were processed using QIIME (version 1.17) software. Operational taxonomic units (OTUs) were clustered with 97% identity cutoff using UPARSE (version 7.1, http://drive5.com/uparse/), and chimeras were determined and removed using Chimer-UCHIME. Representative sequences of each OTU were classified with a 90% confidence level using Silva release 132 (http://www.arb-silva.de). Alpha Diversity was used to analyze the complexity of species diversity for a sample through four indices. Ace and Chao richness estimators, and Shannon and inverse Simpson diversity indices were conducted using the MOTHUR program [29]. Higher values of Ace index and Chao index represent more richness. A higher value of Shannon index represents more diversity, while a higher value of Simpson index represents less diversity. Beta diversity analysis was used to evaluate differences in overall bacterial community composition among different samples, unweighted Unifrac principal coordinate analysis (PCoA, Beta diversity) was performed based on Bray–Curtis distance [30], evaluating the dissimilarities amongst the specimens. A distance-based analysis of molecular variance (AMOVA) was used to assess the significant differences between AH02LA and control groups [29]. The sequence information was submitted to the GenBank Sequence Read Archive database under accession number SRP 174443.

### 2.6. SCFA and Lactate Concentrations Analysis in the Ileum and Colon

Ileal and colonic digesta were used for analyzing SCFA using gas chromatography as described previously [31]. Lactate concentrations in each sample were measured using assay commercial kit (Nanjing Jiancheng Biological Engineering Institute, Nanjing, China).

### 2.7. Statistical Analysis

Data were analyzed using SPSS 20.0 software (SPSS Inc., Chicago, IL, USA). The data of microbiota, microbial metabolites, and immune markers were analyzed using the Mann–Whitney *U* test corrected with the false-discovery rate (FDR). Data were expressed as mean ± SEM and significance was set at *p* ≤ 0.05. Correlation analysis between immune markers or bacterial fermentation products with bacteria was analyzed by Pearson’s correlation analysis using GraphPad Prism version 5.00 (GraphPad Software, San Diego, CA, USA).

## 3. Results

### 3.1. Pseudorabies Virus (PRV) DNA Detection in the Ileal and Colonic Mucosa

All pigs in the AH02LA group developed typical clinical symptoms (anorexia, spirits atrophy, and neurologic symptoms) since day 3 post inoculation. Rectal temperature is shown in Figure S1. At day 3 post infection, all pigs in AH02LA group displayed fever with rectal temperature between 40.5 and 42.0 °C. Three pigs in the AH02LA group showed diarrhea. Successful PCR amplification of 18S rRNA gene (positive control) from ileal and colonic mucosa and brain and lung DNA was shown in Figure S2. Virus DNA was detected in brain and lung in AH02LA group (Figure S3), suggesting that the challenge was successfully achieved. However, no virus was detected in the ileal and colonic mucosa of the piglets in either AH02LA or control groups by PCR (Figure S4) and real-time qPCR analysis (below the detection limit).

### 3.2. Gene Expression in Ileal and Colonic Mucosa

**Figure 1:**
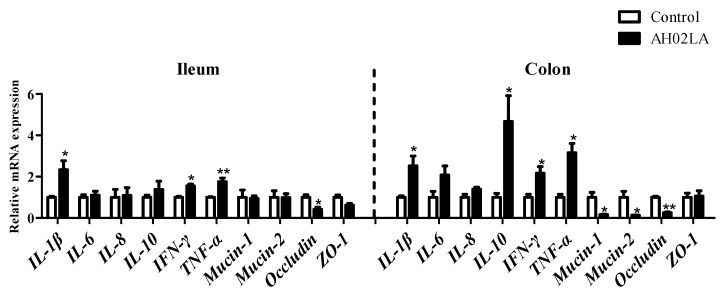
Effects of intranasal pseudorabies virus (PRV) AH02LA strain on the mRNA expression of genes related to cytokines and barrier function in the ileal and colonic mucosa of piglets at day 5 post intranasal infection. The values are expressed as the means ± SEM (*n* = 5). Asterisks indicate statistically significant difference from control. * *p* < 0.05, ** *p* < 0.01. The *β*-actin mRNA level was used to normalize the relative amount of each studied mRNA, and the2^−^^ΔΔ^^Ct^ method was used to analyze the data. *IL*, interleukin; *IFN-γ*, interferon-γ; *TNF-α*, tumor necrosis factor-α; *ZO-1*, zonula occludens-1.

Immune-related gene expression in the ileal and colonic mucosa of piglets with AH02LA and control groups are shown in Figure 1. For cytokines, in the ileal mucosa, AH02LA infection markedly up-regulated (*p* < 0.05) gene expression of *IL-1β*, *IFN-γ*, and *TNF-α*. However, mRNA expression levels of *IL-6*, *IL-8*, and *IL-10* were similar in both groups. In the colonic mucosa of piglets infected with PRV AH02LA, *IL-1β*, *IL-10*, *IFN-γ*, and *TNF-α* mRNA expression levels were higher than control piglets (*p* < 0.05), while *IL-6* and *IL-8* mRNA expression levels did not differ significantly between the AH02LA and control groups. For intestinal barrier-related gene (*Mucin-1*, *Mucin-2*, *Occludin* and *ZO-1*), in the ileal mucosa, PRV AH02LA infection down-regulated (*p* < 0.05) *Occludin* gene expression, but had no impact on *Mucin-1*, *Mucin-2*, and *ZO-1* gene expression. In the colonic mucosa of piglets infected with PRVAH02LA, mRNA expression levels of *Mucin-1*, *Mucin-2*, and *Occludin* were lower (*p* < 0.05), whereas mRNA expression level of *ZO-1* was not significantly different between AH02LA and control groups. These data indicate that PRV AH02LA strain up-regulated specific cytokines mRNA expression, but down-regulated specific intestinal barrier-related gene expression in the ileum and colon at day 5 post intranasal infection. Furthermore, we observed up-regulation of specific cytokines gene expression (*IL-1β*, *IFN-γ* and *TNF-α* in the ileum; *IFN-γ* and *TNF-α* in the colon), down-regulation of intestinal barrier-related gene expression (*Mucin-2* in the colon) in piglets with diarrhea compared to piglets without diarrhea (Figure S5). 

### 3.3. sIgA Concentration in the Ileum and Colon

**Figure 2:**
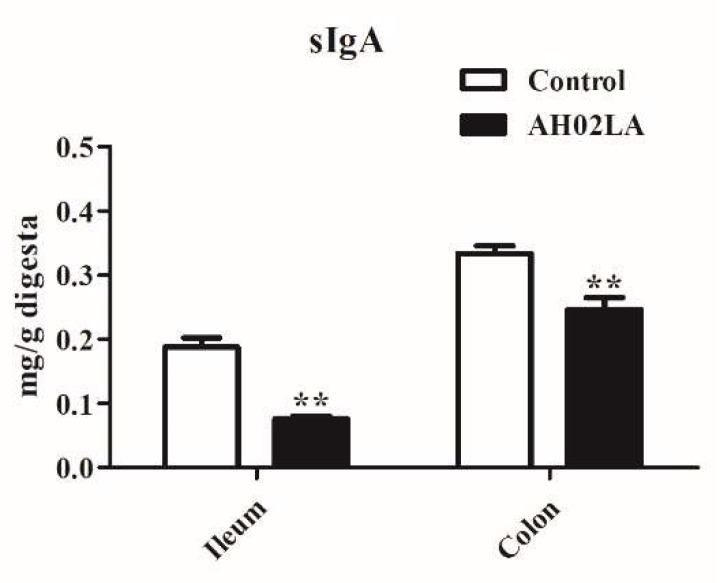
Effects of intranasal pseudorabies virus (PRV) AH02LA strain on the concentration of secretory immunoglobulin A (sIgA) in the ileal and colonic digesta of piglets at day 5 post intranasal infection. The values are expressed as the means ± SEM (*n* = 5). Asterisks indicate statistically significant difference from control. ** *p* < 0.01.

As shown in Figure 2, PRV AH02LA strain significantly decreased (*p* < 0.05) sIgA concentration in the ileal and colonic digesta at day 5 post intranasal infection. In the ileum and colon of piglets with diarrhea, the concentration of sIgA was lower than piglets without diarrhea (*p* < 0.05, Figure S6).

### 3.4. Bacterial Community in the Ileal and Colonic Digesta

**Figure 3:**
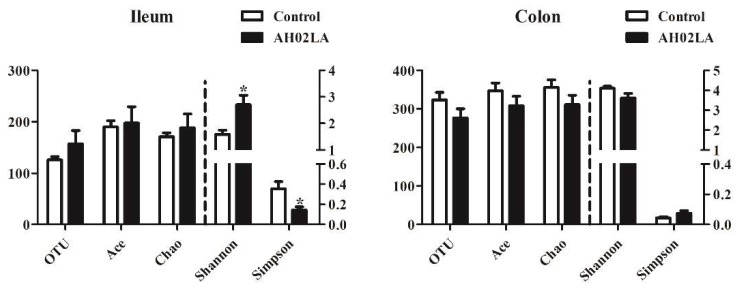
Diversity of ileal and colonic bacterial community in piglets of control and AH02LA groups. The values are expressed as the means ± SEM (*n* = 5). Asterisks indicate statistically significant difference from control: * *p* < 0.05.

**Figure 4:**
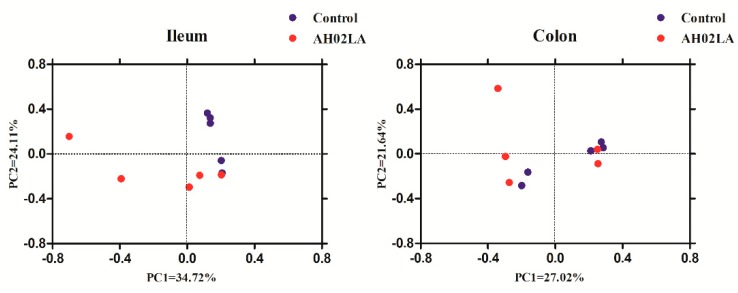
Principal coordinate analysis (PCoA) of ileal and colonic bacterial communities in piglets of control and AH02LA groups by Bray–Curtis similarity metric. PC, principal coordinate.

Ileal and colonic bacterial community compositions in control and AH02LA groups were revealed by Illumina MiSeq sequencing of 16S rRNA. In total, 643,245 V3–V4 16S rRNA gene reads from 20 samples with 32,162 sequences per sample were obtained for further analysis. The bacterial richness and diversity indices are shown in Figure 3. In the ileum, PRV AH02LA infection increased (*p* < 0.05) species diversity indices, resulting in an increase in Shannon index and a decrease in Simpson index as compared to that in the control group. However, in the colon, species richness (ACE and Chao) and diversity indices (Simpson and Shannon) did not differ between the AH02LA and control groups. Furthermore, in the ileum, species diversity indices were higher (an increase in Shannon index and a decrease in Simpson index) in piglets with diarrhea compared to piglets without diarrhea (*p* < 0.05, Figure S7). For β-diversity (Figure 4), PCoA result revealed that axis 1 accounted for 34.72% (ileum) and 27.02% (colon) of the variation, and axis 2 for 24.11% and 21.64% of the variation, respectively. In addition, AMOVA analysis indicates that bacterial communities in the ileum were significantly affected by PRV AH02LA infection (*Fs* = 2.13, *p* = 0.016). However, colonic samples from both groups were not separated.

**Figure 5:**
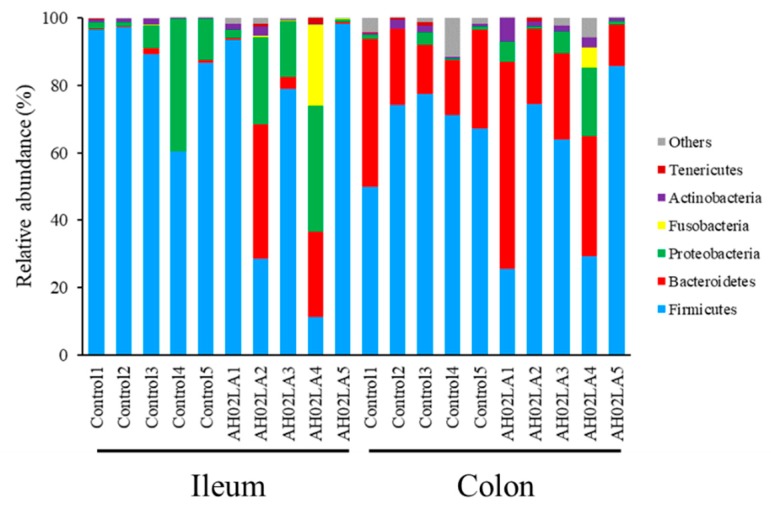
Ileal and colonic bacterial composition profiles at the phylum in piglets of control and AH02LA groups.

At the phylum level, Firmicutes (74.04% ± 9.78% in the ileum and 61.88% ± 6.46% in the colon), Bacteroidetes (7.25% ± 4.38% in the ileum and 28.33% ± 4.78% in the colon) and Proteobacteria (14.49% ± 4.71% in the ileum and 4.14% ± 1.95% in the colon) were the 3 predominant phyla in the ileum and colon (Figure 5). Fusobacteria (2.49% ± 2.37% in the ileum and 0.61% ± 0.61% in the colon) and Actinobacteria (0.90% ± 0.28% in the ileum and 2.00% ± 0.61% in the colon) constituted the next 2 phyla in the ileum and colon. No significant difference was observed for the relative abundance of bacterial phyla between AH02LA and control groups. In the ileum, the relative abundance of Firmicutes significantly decreased (*p* < 0.05) in piglets with diarrhea compared to piglets without diarrhea, but the abundance of Bacteroidetes increased (*p* < 0.05, Figure S8).

**Figure 6:**
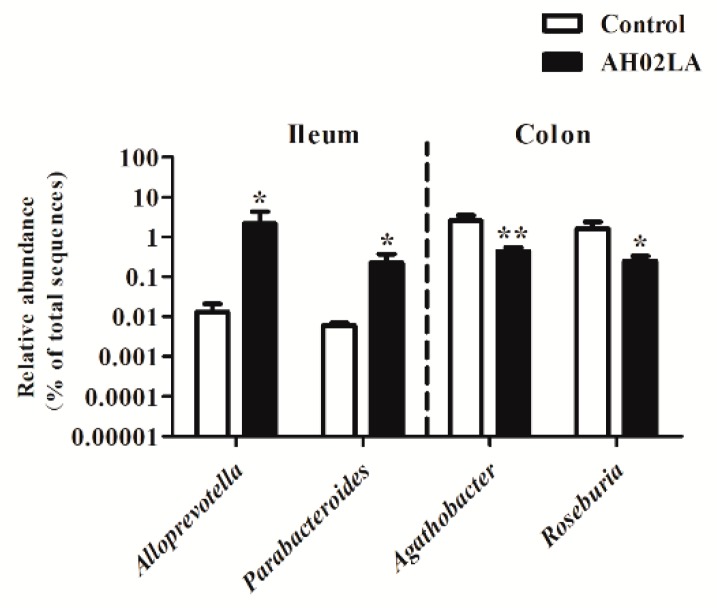
Significantly changed genera in the ileal and colonic digesta in piglets of control and AH02LA groups. Values are means ± SEMs (*n* = 5). Asterisks indicate statistically significant difference from control (Mann–Whitney *U* test and a false discovery rate). * *p* < 0.05, ** *p* < 0.01.

The top 30 bacterial genera in the ileum and colon are listed in Supplementary Figure S9. In the ileum, *Lactobacillus*, *Streptococcus*, *Escherichia-Shigella*, unclassified Caulobacteraceae, *Fusobacterium,* and *Bacteroides* were the most abundant genera (>2%). The relative abundance of *Alloprevotella and Parabacteroides* was higher (*p* < 0.05) in the AH02LA group compared to the control group (Figure 6). In the colon, the dominant genera (>2%) were *Lactobacillus, Alloprevotella,* Unclassified Muribaculaceae, Prevotellaceae NK3B31 group, Ruminococcaceae UCG-014, *Prevotella* 9, Ruminococcaceae UCG-005, Lachnospiraceae NK4A136 group, and *Desulfovibrio*. PRV AH02LA infection decreased (*p* < 0.05) the abundance of *Agathobacter* and *Roseburia* (Figure 6) as compared with control group. Furthermore, in the ileum, the relative abundance of *Fusobacterium*, *Bacteroides*, *Campylobacter*, *Butyricimonas*, *Desulfovibrio*, *Parabacteroides*, *Alistipes*, *Candidatus Soleaferrea,* and *Parasutterella* were higher (*p* < 0.05) in piglets with diarrhea compared to piglets without diarrhea (Figure S10). *Sharpea* was solely detected in the piglets with diarrhea. In the colon, piglets with diarrhea resulted in a higher abundance of *Enterococcus*, and a lower abundance of *Agathobacter* (*p* < 0.05). *Prevotella* 7 was solely detected in piglets without diarrhea, and *Oxalobacter* was solely detected in piglets with diarrhea.

**Figure 7:**
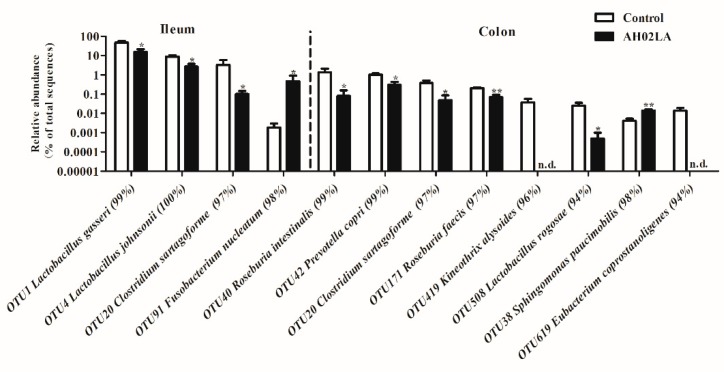
Significantly changed bacteria operational taxonomic units (OTUs) in the ileal and colonic digesta of piglets in control and AH02LA groups. Values are means ± SEMs (*n* = 5). Asterisks indicated statistically significant difference from control (Mann–Whitney *U* test and a false discovery rate). * *p* < 0.05, ** *p* < 0.01. n.d., no sequence detected.

The top 30 dominant phylotypes (OTUs) in the ileum and colon are listed in Supplementary Figure S11. In the ileum, the relative abundance of OTUs closely related to *Lactobacillus gasseri*, *Lactobacillus johnsonii* and *Clostridium sartagoforme* were lower (*p* < 0.05) in the AH02LA group compared to the control group (Figure 7). Moreover, PRV AH02LA infection increased (*p* < 0.05) the abundance of OTU 91 (*Fusobacterium nucleatum*). In the colon, the abundance of several OTUs belonging to *Lactobacillus reuteri*, *Roseburia hominis*, *Roseburia intestinalis*, *Prevotella copri*, *Clostridium sartagoforme*, *Roseburia faecis,* and *Lactobacillus rogosae* was lower (*p* < 0.05) in the AH02LA group than that in the control group, whereas the abundance of OTU38 (*Sphingomonas paucimobilis*) was higher in the AH02LA group compared with control group (*p* < 0.05). The OTUs related to *Kineothrix alysoides* and *Eubacterium coprostanoligenes* were solely detected in the control group. Furthermore, in the ileum, the abundance of OTUs closely related to *Actinobacillus porcinus*, *Fusobacterium nucleatum*, *Bacteroides thetaiotaomicron*, *Campylobacter fetus*, *Desulfovibrio piger*, *Agathobacter ruminis*, *Parabacteroides johnsonii,* and *Lactococcus piscium* were higher (*p* < 0.05) in piglets with diarrhea compared to piglets without diarrhea (Figure S12). The OTUs related to *Butyricimonas virosa*, *Sharpea azabuensis*, *Alistipes senegalensis* and *Tyzzerella nexilis* were solely detected in the piglets with diarrhea.

In the colon, the abundance of OTUs closely related to *Roseburia intestinalis*, *Lactobacillus antri*, *Prevotella copri* and *Catenibacterium mitsuokai* significantly decreased (*p* < 0.05) in piglets with diarrhea compared to piglets without diarrhea, whereas the abundance of OTUs belonging to *Enterococcus cecorum* and *Bacteroides stercoris* increased (*p* < 0.05).

Taken together, these results suggest that intranasal PRV AH02LA strain affected intestinal bacteria, resulting in a decrease in some generally beneficial bacteria (*Lactobacillus* species in the ileum and colon; butyrate-producing bacteria in the colon) and an increase in potentially pathogenic *Fusobacterium nucleatum* in the ileum and *Sphingomonas paucimobilis* in the colon at day 5 post intranasal infection. 

### 3.5. Short-Chain Fatty Acids (SCFA) and Lactate Concentrations in Ileal and Colonic Digesta 

**Figure 8:**
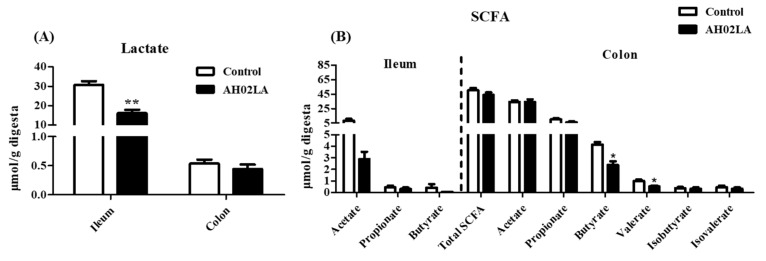
Effects of intranasal pseudorabies virus (PRV) AH02LA strain on the concentrations of lactate (**A**) and SCFA (**B**) in the ileal and colonic digesta of piglets at day 5 post intranasal infection. The values are expressed as the means ± SEM (*n* = 5). Asterisks indicate statistically significant difference from control. * *p* < 0.05, ** *p* < 0.01. SCFA, short-chain fatty acid.

Lactate, which is produced from carbohydrate fermentation (mainly in the small intestine) by lactic acid-producing bacteria, such as *Lactobacillus* and *Bifidobacterium*, can prevent proliferation of pathogenic bacteria and maintain gut health. In this study, PRV AH02LA infection significantly decreased (*p* < 0.05) lactate concentration in the ileum, but had little effect on lactate concentration in the colon (Figure 8A). SCFAs are measured as indicators of microbial fermentation in the gut, particularly the hind gut. In this study, the concentration of SCFAs in the ileum were low and PRV AH02LA infection had no significant effects on the concentrations of acetate, propionate, or butyrate (Figure 8B). However, valerate, isobutyrate, and isovalerate were below the limit of detection in both groups. In the colon, PRV AH02LA infection significantly decreased the concentrations of butyrate and valerate (*p* < 0.05), but only slightly reduced the propionate concentration (*p* = 0.08). Furthermore, the concentrations of lactate in the ileum and butyrate in the colon significantly decreased (*p* < 0.05) in piglets with diarrhea compared to piglets without diarrhea (Figure S13).

### 3.6. Correlation Analysis of Immune Markers, Bacteria, and Microbial Fermentation Products in the Ileum and Colon and Body Temperature 

**Figure 9:**
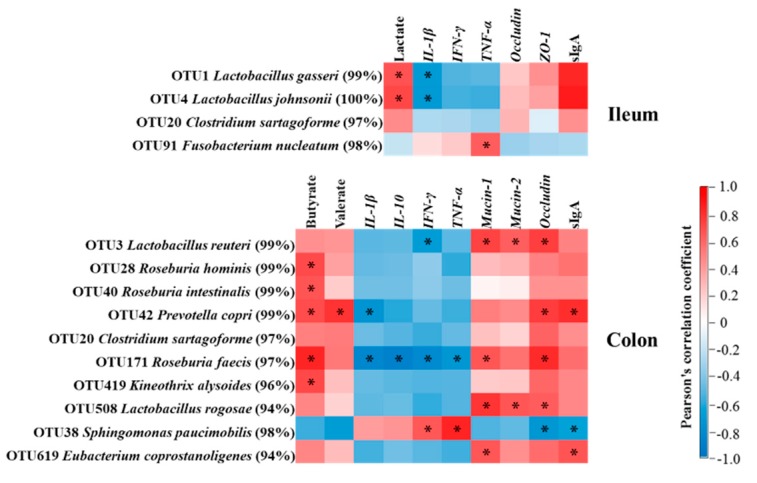
Correlation analysis between bacteria with immune markers or bacterial fermentation products in the ileum and the colon. The red represents a significant positive correlation, and the blue represents a significant negative correlation. * *p* < 0.05. *IL*, interleukin; *IFN-γ*, interferon-γ; *TNF-α*, tumor necrosis factor-α; *ZO-1*, zonula occludens-1; sIgA, secretory immunoglobulin A.

Correlation between immune markers or bacterial fermentation products with bacteria in the ileum and colon are shown in Figure 9. In the ileum, the concentrations of sIgA and lactate positively correlated (*p* < 0.05) with the abundance of OTUs related to *Lactobacillus gasseri* and *Lactobacillus johnsonii.* The mRNA expression level of *TNF-α* positively correlated (*p* < 0.05) with the abundance of OTU related to *Fusobacterium nucleatum*. In the colon, the concentration of butyrate positively correlated (*p* < 0.05) with the abundance of OTUs related to *Roseburia hominis*, *Roseburia intestinalis*, *Prevotella copri*, *Roseburia faecis,* and *Kineothrix alysoides*. The mRNA expression levels of *IL-1β*, *IL-10*, *IFN-γ*, and *TNF-α* negatively correlated (*p* < 0.05) with the abundance of OTU related to *Roseburia faecis.* The mRNA expression levels of *IFN-γ* and *TNF-α* positively correlated (*p* < 0.05) with the abundance of OTU related to *Sphingomonas paucimobilis*. The mRNA expression levels of *Mucin-1*, *Mucin-2*, and *Occludin* positively correlated (*p* < 0.05) with the abundance of OTUs related to *Lactobacillus reuteri* and *Lactobacillus rogosae*. The mRNA expression levels of *Mucin-1* and *Mucin-2* were positively correlated (*p* < 0.05) with the abundance of OTU related to *Roseburia faecis*. *Occludin* mRNA expression level and sIgA concentration were positively correlated (*p* < 0.05) with the abundance of OTU related to *Prevotella copri*, but negatively correlated (*p* < 0.05) with the abundance of OTU related to *Sphingomonas paucimobilis*. These results indicate that the changes in metabolites or immune markers correlated with alterations of bacteria in the ileum and colon of pigs.

Correlation between immune markers, bacteria, or bacterial fermentation products in the ileum and colon with rectal temperature are shown in Figure S14. For the ileum, rectal temperature was positively correlated with the mRNA expression levels of *IL-1β*, *IFN-γ*, and *TNF-α*, but negatively correlated with the concentrations of sIgA and lactate, the mRNA expression level of Occludin, and the abundance of OTUs related to *Lactobacillus gasseri* and *Lactobacillus johnsonii*. For the colon, rectal temperature positively correlated with the mRNA expression levels of *IL-1β*, *IFN-γ*, and *TNF-α* and the abundance of OTU related to *Sphingomonas paucimobilis*, but negatively correlated with the concentrations of sIgA, butyrate, and valerate, the mRNA expression levels of *Mucin-1*, *Mucin-2*, and *Occludin* and the abundance of OTUs related to *Prevotella copri*, *Roseburia faecis,* and *Eubacterium coprostanoligenes.* These results indicate that the changes in bacteria, metabolites, or immune markers in the ileum and colon correlated with alterations of body temperature.

## 4. Discussion

Pseudorabies is a devastating disease in the livestock industry, and causes fever, respiratory distress, systemic neurological symptoms, diarrhea, and high mortality in piglets [3]. The effects of PRV on nervous system and respiratory system have been widely investigated [32,33]. To our knowledge, this was the first study to analyze the response of ileal and colonic bacteria community and immunity in piglets in response to intranasal PRV infection. We demonstrate that PRV AH02LA strain negatively affected gut homeostasis by altering bacterial community and some immune markers in the ileum and colon, and decreasing the lactate in the ileum and butyrate in the colon at day 5 post intranasal infection. The findings provide insight into the impact of intranasal PRV infection on gut immunity and bacterial community in piglets.

### 4.1. Intranasal Pseudorabies Virus (PRV) AH02LA Infection Influences Some Immune Markers in the Ileum and Colon

Previous studies have shown that intranasal PRV infection upregulates cytokine mRNA expression in the nervous system (trigeminal ganglion and brain stem) in 2-week-old piglets and the respiratory system (nasal mucosa) in 15-week-old swine [6]. However, little is known regarding the impact of intranasal PRV infection on intestinal immunity in piglets. Cytokines play a crucial role in the immune and inflammatory responses, and their balance is important to block virus replication and prevent clinical disease [34]. Our data showed that PRV AH02LA infection upregulated specific cytokines expression, such as *IL-1β*, *IFN-γ,* and *TNF-α* in the ileum and *IL-1β*, *IL-10*, *IFN-γ*, and *TNF-α* in the colon (Figure 1). *IFN-γ*, which possesses antiviral activity and acts as a potent activator of macrophages, produces proinflammatory cytokines such as *TNF-α*. The increased cytokines levels suggest that intranasal PRV AH02LA infection affected intestinal immune response. Epithelial cells form the intestinal barrier via tight junctions, which plays an important role in preventing pathogenic agents and luminal antigens [35]. The current study showed that intranasal PRV AH02LA infection down-regulated intestinal barrier genes expression in the ileum and colon, including *Mucin-1*, *Mucin-2*, and *Occludin* (Figure 1). This suggests that intranasal PRV AH02LA infection decreases the integrity of the mucous layer, which might become susceptible to pathogen infection. sIgA plays an integral role in the protection of the intestine against pathogens invading the intestinal mucosa [36]. The lower concentration of intestinal sIgA in the ileum and colon of AH02LA group (Figure 2) might indicate a relatively weakened defensive capacity. However, the potential mechanisms behind mucosal immune responses is unknown. We did not detect AH02LA in the ileum and colon suggesting that the mucosal immune responses were not directly induced by virus propagation. Previous studies demonstrated cross-talks between intestinal immunity with serum immunity or lung immunity [37,38]. In a mouse model of respiratory influenza infection, the lymphocytes derived from the lung respiratory mucosa migrated into the intestinal mucosa and actively secreted *IFN-γ* in the absence of virus antigen stimulation [38]. The changes in ileal and colonic immune index in response to intranasal PRV AH02LA infection may be attributed to the migration of inflammatory cytokines and cells, as opposed to direct virus antigen stimulation.

### 4.2. Intranasal Pseudorabies Virus (PRV) AH02LA Infection Alters the Microbiota and Their Metabolites in the Ileum and Colon

Bacterial communities play an essential role in maintaining the homeostasis of the intestinal immune system. We found that PRV AH02LA infection increased species diversity indices by increasing Shannon index while reducing Simpson index (Figure 3), which was also identified by PCoA (Figure 4) and AMOVA analysis. These results were inconsistent with previous finding, in which PEDV and influenza virus infection reduced microbiome diversity [14,39]. Intestinal immunity plays an important role in regulating and controlling bacterial composition [40]. During virus infection, the immune function of the host gradually changes. In the present study, intestinal immune function unbalance in the piglets of AH02lA at the stage of morbidity (day 5 after infection) might have led to the increasing species diversity in the ileum. Elucidating the time-course effects of intranasal PRV AH02LA infection on intestinal immunity and bacterial composition requires further investigation. Actually, the acute infection period of PRV lasts for 3~10 days, and PRV usually establishes a life-long latent infection in the host peripheral nervous system and lymphoid tissues post-acute infection [1]. Therefore, it is difficult to find pigs with PRV completely cleared following acute infection, and to investigate the intestinal flora status of such pigs. However, the intestinal flora and immune status of pigs after recovery from acute infection are important for the elucidation of relationship between PRV infection and intestinal flora, and thus will be our next research content.

In addition to microbial diversity, differences in the relative abundance of OTUs were detected between control and AH02LA groups. PRV AH02LA infection decreased the abundance of bacteria with beneficial function and increased several potential pathogens in the ileum and colon. For example, *Lactobacillus gasseri* and *Lactobacillus johnsonii* in the ileum and *Lactobacillus reuteri*, *Roseburia hominis*, *Roseburia intestinalis*, *Roseburia faecis, Lactobacillus rogosae,* and *Kineothrix alysoides* in the colon were markedly decreased in AH02LA group (Figure 7). *Lactobacillus* species (*Lactobacillus gasseri*, *Lactobacillus johnsonii, Lactobacillus reuteri,* and *Lactobacillus rogosae*) can inhibit pathogens colonization, attenuate viral infection, and produce antimicrobial substances (bacteriocins and lactic acid), and are thus regarded as potentially beneficial species [41,42,43]. Butyrate-producing bacteria (*Roseburia hominis*, *Roseburia intestinalis*, *Roseburia faecis,* and *Kineothrix alysoides*) play an important role in maintaining gut homeostasis and modulating immune development [44,45]. Furthermore, PRV AH02LA infection increased the relative abundance of *Fusobacterium nucleatum* in the ileum and *Sphingomonas paucimobilis* in the colon (Figure 7). As a microbial pathogen, *Fusobacterium nucleatum* contributes to the initiation and progression of intestinal cancer [46]. *Sphingomonas paucimobilis* is an opportunistic pathogen that causes infections in immunocompromised and hospitalized patients [47]. Taken together, these data point to the potentially negative effect of intranasal PRV infection on gut microbial community in piglets. 

The present study showed that PRV AH02LA infection significantly decreased lactate concentration in the ileum (Figure 8A). Lactate in the intestine results from bacterial production, host absorption, and microbial utilization. *Lactobacillus* is considered as the main lactate producer in the intestine. A positive correlation between lactate concentration and the abundance of OTUs related to *Lactobacillus gasseri and Lactobacillus johnsonii* in the ileum (Figure 9) indicates that the reduction in the abundance of *Lactobacillus gasseri* and *Lactobacillus johnsonii* might partially contribute to the decreased lactate concentration in the ileum of AH02LA group. High level of lactate in the small intestine is beneficial to gut health by decreasing intestinal pH and delaying the multiplication of an enterotoxigenic *E. coli* [11]. The decreased lactate concentration following intranasal PRV AH02LA infection might partly make the small intestine more vulnerable to pathogens. SCFAs (acetate, propionate, and butyrate), the main products of microbial carbohydrate fermentation, have beneficial effects on gut functions. Our findings showed that PRV AH02LA infection decreased butyrate and valerate concentrations in the colon (Figure 8B). Butyrate not only exerts anti-inflammatory functions, but is a major energy source for intestinal epithelial cells [48]. The role of valerate on gut function is currently unclear and needs further investigation. In this study, butyrate concentration positively correlated with the abundance of OTUs related to *Roseburia hominis*, *Roseburia intestinalis*, *Roseburia faecis*, and *Kineothrix alysoides* (Figure 9). Decreased butyrate in the AH02LA group may be attributed to the reduction of butyrate-producing bacteria. 

### 4.3. Bacterial Composition Change Are Associated with Altered Immune Markers and Body Temperature

The mechanism involved in the changes of the gut microbiota in response to intranasal PRV AH02LA infection remain unclear. Nervous and immune system exert critical control over microbiota composition [10,49]. In the current study, intestinal immunity unbalance due to PRV infection might be one of the mechanisms by which intranasal PRV AH02LA infection resulted in changes in the composition of the gut microbiota. A previous study has shown that increased *IL-22* level with *Salmonella* *Typhimurium* infection liberates a colonization niche for the pathogenic bacteria by suppressing commensal bacteria in mice [50]. Moreover, influenza-induced *IFN-Is* promote the depletion of obligate anaerobic bacteria and the enrichment of Proteobacteria in the gut, leading to a dysbiotic micro-environment in mice [51]. Our correlation results showed that the change of cytokine mRNA expression levels positively correlated with the abundance of potentially pathogenic bacteria, but negatively correlated with that of generally beneficial bacteria (Figure 9), indicating a potential causal relationship between the increased cytokines expression and the change in bacterial community. Furthermore, the abundance of OTU related to *Sphingomonas paucimobilis* was negatively correlated with sIgA concentration (Figure 9). sIgA is important for the maintenance of diversity and composition of microbiota [52]. The decreased sIgA concentration might partly led to a failure to suppress the growth of *Sphingomonas paucimobilis*. Thus, the observed change in bacterial community after intranasal PRV AH02LA infection provide hypotheses for virus–bacterial interactions mediated by the immune system.

Furthermore, clinical symptoms (such as fever and anorexia) may partly affect gut homeostasis. Correlation results showed that the changes in bacteria, metabolites, or immune markers in the ileum and colon are correlated with alterations of body temperature (Figure S14). Fever in infected pigs may affect intestinal physiological function (such as digestive enzyme activity), inducing the change of intestinal microbiota and immune status. Diet is the biggest contributor to gut health. The change in food or water intake in infected pigs with high fever, depression, respiratory distress, or systemic neurological symptoms may also result in alteration of intestinal microbiota and immune status. In the future study, the infected pigs and non-infected pigs fed the same diet with limited food and water are necessary to better explain the change in intestinal microbiota and immune status.

It is well known that PRV causes diarrhea in piglets. However, the potential mechanisms remain unclear. We initially thought that diarrhea and gut immunopathology are induced by viral replication in the gut. However, it is surprising for us to find out that PRV was not detected in the mucosa of the ileum or the colon of pigs at day 5 post intranasal infection in this study (Figure S4). The observed changes of gut homeostasis might be a direct effect of the intranasal PRV AH02LA infection. The viral infection stimulated the production of inflammatory cytokines and cells in other tissue, which might then migrate to the ileum and colon and induce the local immune changes, consequently disturbing the gut microbiota. Alternatively, the changes in gut microbiota and immune status after intranasal PRV AH02LA infection in our study might be associated with infection-induced fever, clinical symptoms and even changes in diet. Furthermore, although differences between pigs with and without diarrhea seem to suggest that the change in food or water intake in infected pigs plays an important role for the alteration of intestinal microbiota and immune status, the sample size is too limited for a statistical analysis (3 pigs with diarrhea in the PRV group, high level of variance in pig intestinal microbial community composition among individuals). In future study, we will compare differences between pigs with and without diarrhea in a much larger sample size. Therefore, in this study, it is difficult to evaluate whether the observed changes are directly due to the PRV infection or rather to indirect effects such as fever, clinical signs, and changes in diet in infected pigs. Further investigation is warranted. 

## 5. Conclusions

PRV AH02LA strain negatively influences microbial composition and immune status in the ileum and colon towards an unhealthy gut environment at day 5 post intranasal infection. PRV AH02LA infection greatly affected the intestinal immunity, as evidenced by upregulation of cytokines expression, downregulation of intestinal barrier-related gene expression, and reduction of sIgA concentration in the ileum and colon. However, no PRV was detected in the ileum and colon. The mechanism behind mucosal immune responses needs further investigation. PRV AH02LA infection altered bacterial composition by decreasing the abundance of potentially beneficial species (*Lactobacillus* species in the ileum and colon and butyrate-producing bacteria species in the colon) and increasing several potential pathogens (*Fusobacterium nucleatum* in the ileum and *Sphingomonas paucimobilis* in the colon). Moreover, PRV AH02LA infection reduced concentration of lactate in the ileum and butyrate in the colon. In summary, our findings provide evidence that intranasal PRV infection directly or indirectly brings gut health risks and implications, although no PRV was detected in the ileum and colon.

## Supplementary Materials

The following are available online at https://www.mdpi.com/1999-4915/11/6/518/s1, Figure S1: Rectal temperatures post intranasal infection with pseudorabies virus (PRV) AH02LA stain, Figure S2: Detection of DNA in brain, lung, ileum and colon of piglets, Figure S3: Detection of pseudorabies virus (PRV) in the brain and lung of piglets infected PRV AH02LA, Figure S4: Detection of pseudorabies virus (PRV) DNA in the ileal and colonic mucosa of piglets infected PRV AH02LA, Figure S5: The mRNA expression of genes in the ileal and colonic mucosa of piglets with diarrhea and without diarrhea, Figure S6: The concentration of secretory immunoglobulin A (sIgA) in the ileal and colonic digesta of piglets with diarrhea and without diarrhea, Figure S7: Diversity of ileal and colonic bacterial community in piglets with diarrhea and without diarrhea, Figure S8: Significantly changed phylum in ileal and colonic digesta of piglets with diarrhea and without diarrhea, Figure S9: Heatmap of dominant genus in the ileum and colon of piglets with control and AH02LA groups, Figure S10: Significantly changed genera in the ileal and colonic digesta of piglets with diarrhea and without diarrhea, Figure S11: Heatmap of dominant OTUs in the ileum and colon of piglets with control and AH02LA groups, Figure S12: Significantly changed bacteria OTUs in the ileal and colonic digesta of piglets with diarrhea and without diarrhea, Figure S13: The concentrations of lactate and SCFA in the ileal and colonic digesta of piglets with diarrhea and without diarrhea, Figure S14: Correlation analysis between rectal temperature with immune markers, bacteria or bacterial fermentation products in the ileum and the colon, Table S1: Primers for PCR or quantitative real-time PCR.
