# Supplementary material for: Effects of Intranasal Pseudorabies Virus AH02LA Infection on Microbial Community and Immune Status in the Ileum and Colon of Piglets
Source: Viruses. 2019 Jun 5;11(6):518. doi: 10.3390/v11060518 (PMC6631256; doi:10.3390/v11060518)
Supplement: Supplementary file 1 [file viruses-11-00518-s001.pdf]

## Supplementary material

**Summary**

The supporting information includes 1 supplementary table and 14 supplementary figures.

**Table S1.** Primers for PCR or quantitative real-time PCR

| Primers [1] | Primer sequences (5'-3') | Reference | Annealing temp.(°C) |
|---|---|---|---|
| *Beta actin* F | AGAGCGCAAGTACTCCGTGT | [1] | 60 |
| *Beta actin* R | ACATCTGCTGGAAGGTGGAC | | |
| *GAPDH* F | TTTGCGTCAGTGTCATCG | [2] | 60 |
| *GAPDH* R | TGCTCTGCCTTGGGTAAT | | |
| *18S rRNA* F | CCCACGGAATCGAGAAAGAG | [3] | 60 |
| *18S rRNA* R | TTGACGGAAGGGCACCA | | |
| *IL-1β* F | TGAAGTGCCGCACCCAAAACCT | [1] | 60 |
| *IL-1β* R | CGGCTCCTCCTTTGCCACAATCA | | |
| *IL-6* F | CCCACCACAAATGCCGGCCT | [1] | 60 |
| *IL-6* R | GAGGGAATGCCCGTGGACGG | | |
| *IL-8* F | ACTGGCTGT TGCCTTCTT | [4] | 60 |
| *IL-8* R | CAGTT CTCTTCAAAAATATCTG | | |
| *IL-10* F | GTCCGACTCAACGAAGAAGG | [1] | 60 |
| *IL-10* R | GCCAGGAAGATCAGGCAATA | | |
| *IFN-γ* F | TCCAGCGCAAAGCCATCAGTG | [5] | 60 |
| *IFN-γ* R | ATGCTCTCTGGCCTTGGAACATAGT | | |
| *TNF-α* F | CCACGCTCTTCTGCCTACTGC | [4] | 60 |
| *TNF-α* R | GCTGTCCCTCGGCTTTGAC | | |
| *Mucin-1* F | GGTACCCGGCTGGGGCATTG | [1] | 60 |
| *Mucin-1* R | GGTAGGCATCCCGGGTCGGA | | |
| *Mucin-2* F | CTGCTCCGGGTCCTGTGGGA | [1] | 60 |
| *Mucin-2* R | CCCGCTGGCTGGTGCGATAC | | |
| *Occludin* F | ATGCTTTCTCAGCCAGCG TA | [4] | 60 |
| *Occludin* R | AAG GTTCCATAGCCTCGGTC | | |
| *ZO-1* F | GAGGATGGTCACACCGTGGT | [4] | 60 |
| *ZO-1* R | GGAGGATGCTGTTGTCTCGG | | |
| *gE* part F | GGCTTCCACTCGCAGCTCTT | The study | |
| *gE* part R | CGCACGTCATCACGAAGGAG | | |
| *gE*-Sybr-F | GGTGTTTGCATAATTTTGTGGGTGG | [6] | 60 |
| *gE*-Sybr-R | GAAAGGGCCGCATGGTCTCA | | |

[1] *IL*, interleukin; *IFN-γ*, interferon-γ; *TNF-α*, tumor necrosis factor-α; *ZO-1*, zonula occludens-1.

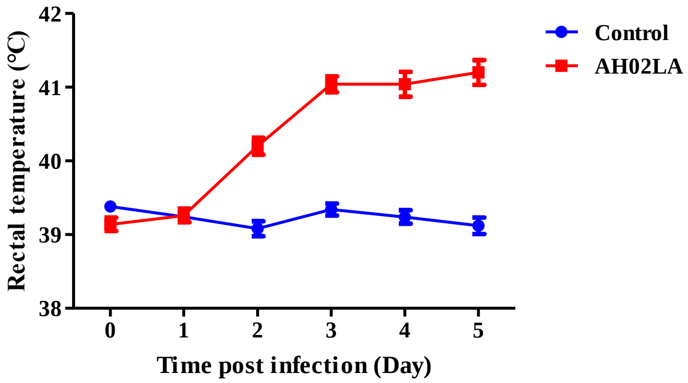


**Figure S1.** Rectal temperatures post intranasal infection with pseudorabies virus (PRV) AH02LA stain. Average temperatures of five piglets of each group were taken for comparison. Error bars represent the standard deviations.

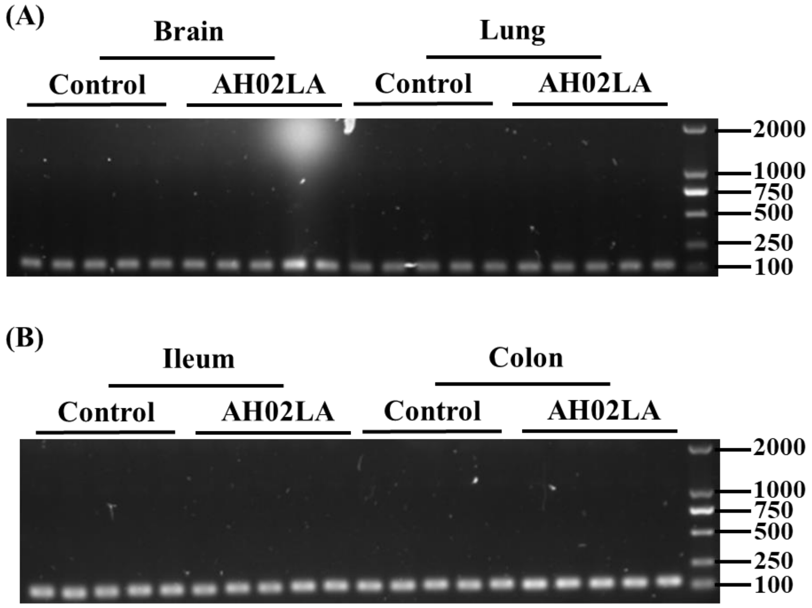


**Figure S2.** Detection of DNA in brain, lung, ileum and colon of piglets. The total DNAs from brain (A), lung (A), ileum (B) and colon (B) were amplified by PCR with a pair of specific primers for *18S rRNA* gene and analyzed by electrophoresis. M: marker DL2000.

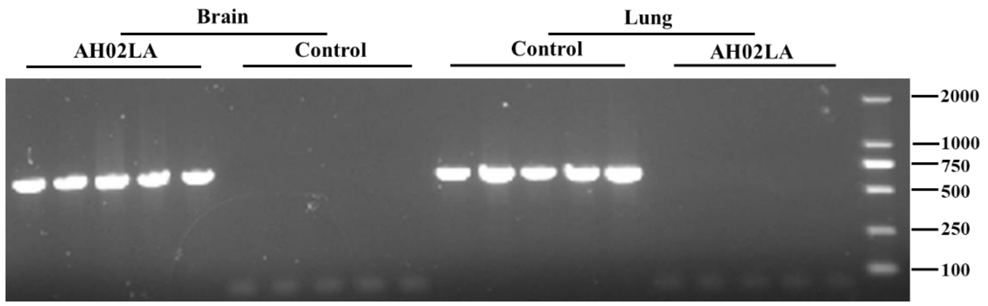


**Figure S3.** Detection of pseudorabies virus (PRV) DNA in the brain and lung of piglets infected PRV AH02LA. The total DNAs from brain and lung were amplified by PCR with a pair of specific primers for *gE* gene and analyzed by electrophoresis. M: marker DL2000.

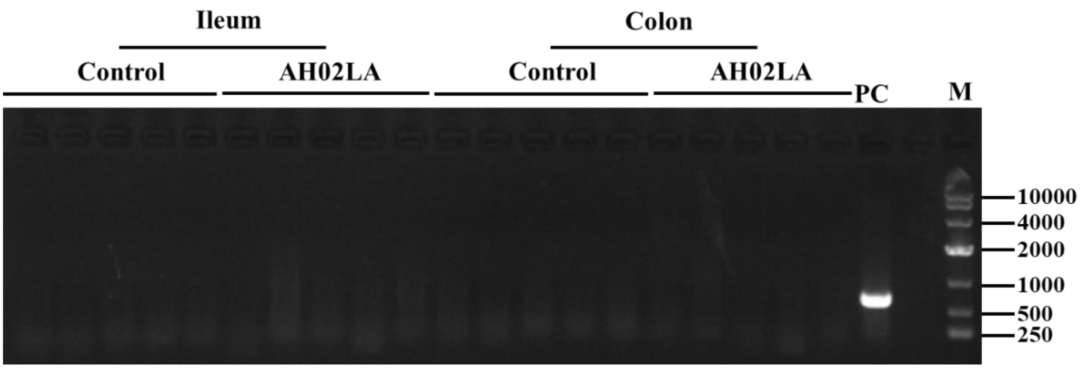


**Figure S4.** Detection of pseudorabies virus (PRV) DNA in the ileal and colonic mucosa of piglets infected PRV AH02LA. The total DNAs from ileal and colonic mucosa were amplified by PCR with a pair of specific primers for gE gene and analyzed by electrophoresis. PC: positive control, DNA from brain of piglets infected AH02LA. M: marker DL10000.

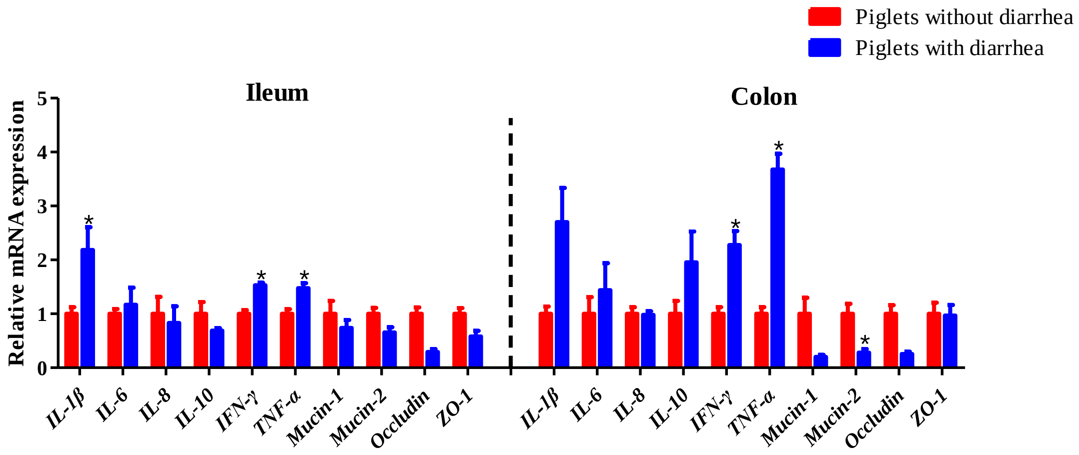


**Figure S5.** The mRNA expression of genes in the ileal and colonic mucosa of piglets with diarrhea (n=3) and without diarrhea (n=7). The values are expressed as the means ± SEM. Asterisks indicate statistical differences between different group. $*P < 0.05$. The β-actin mRNA level was used to normalize the relative amount of each studied mRNA, and the$2^{-\Delta\Delta Ct}$ method was used to analyze the data. *IL*, interleukin; *IFN-γ*, interferon-γ; *TNF-α*, tumor necrosis factor-α; *ZO-1*, zonula occludens-1.

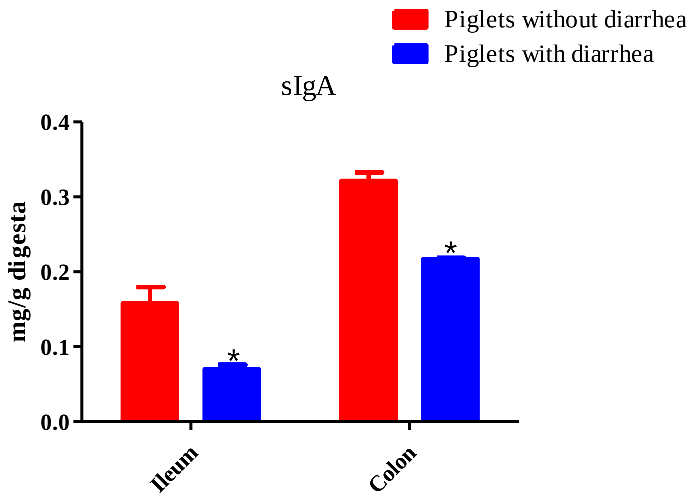


**Figure S6.** The concentration of secretory immunoglobulin A (sIgA) in the ileal and colonic digesta of piglets with diarrhea (n=3) and without diarrhea (n=7). The values are expressed as the means ± SEM. Asterisks indicate statistical differences between different group. *$P < 0.05$.

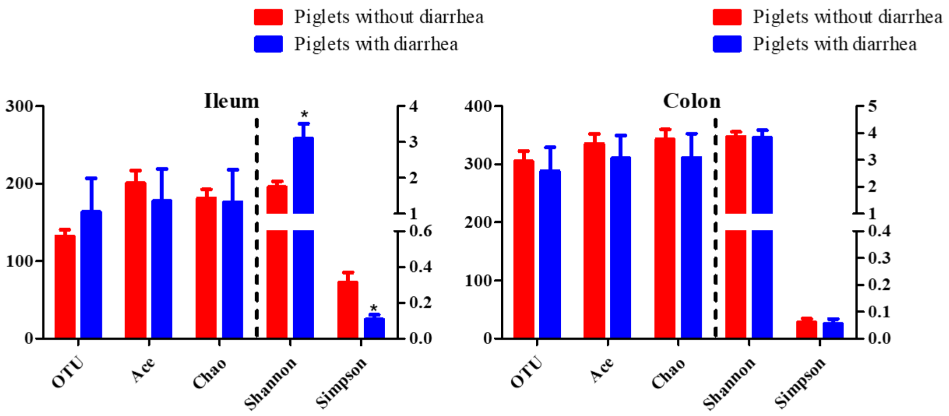


**Figure S7.** Diversity of ileal and colonic bacterial community in piglets with diarrhea (n=3) and without diarrhea (n=7). The values are expressed as the means ± SEM. Asterisks indicate statistical differences between different group. *$P < 0.05$.

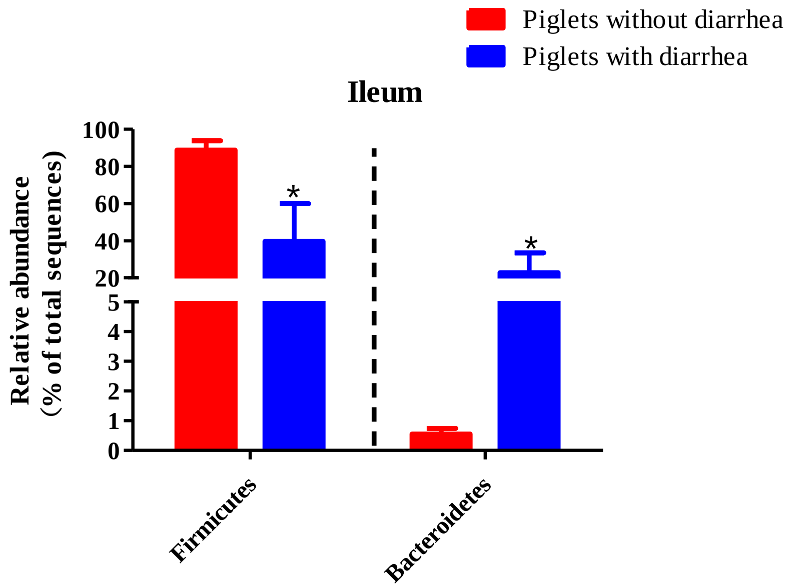


**Figure S8.** Significantly changed phylum in ileal and colonic digesta of piglets with diarrhea (n=3) and without diarrhea (n=7). The values are expressed as the means ± SEM. Asterisks indicate statistical differences between different group. *$P$ < 0.05.

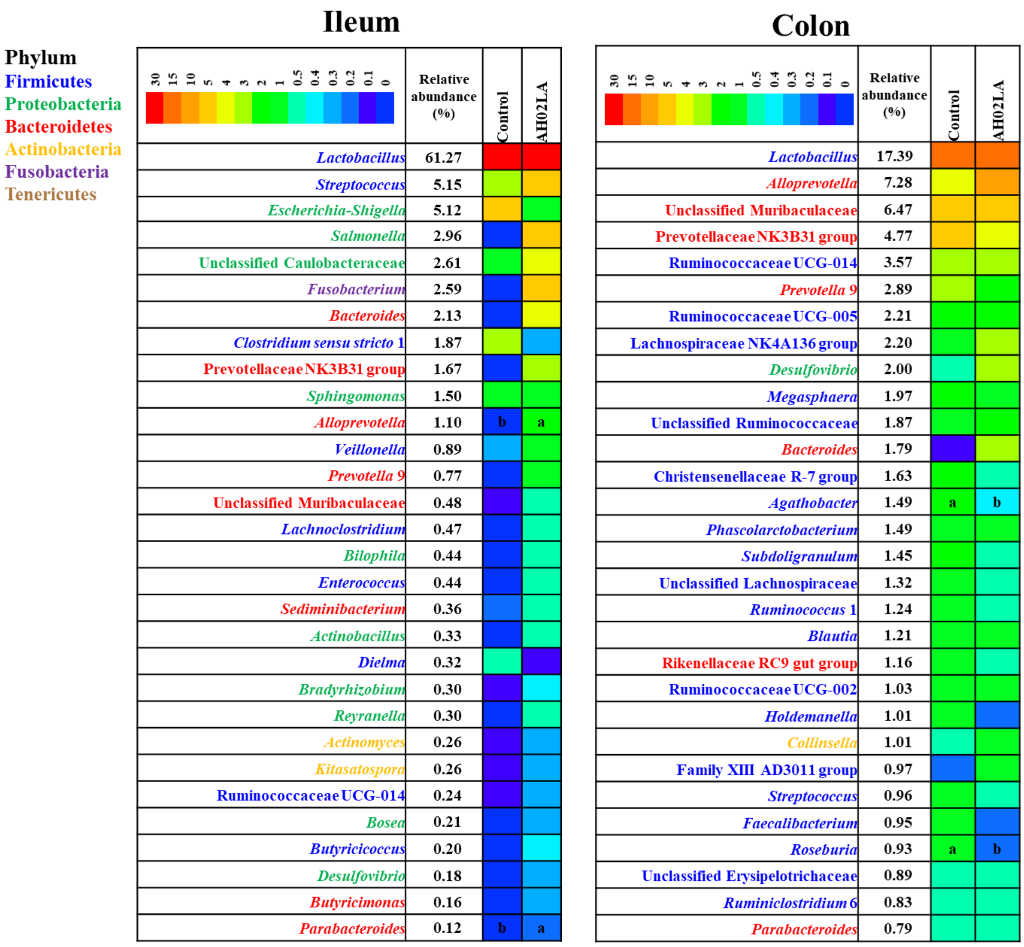


**Figure S9.** Heatmap of dominant genus in the ileum and colon of piglets with control and AH02LA groups. a, b means in a row with different superscripts differ. (Mann–Whitney U test and a false discovery rate < 0.05).

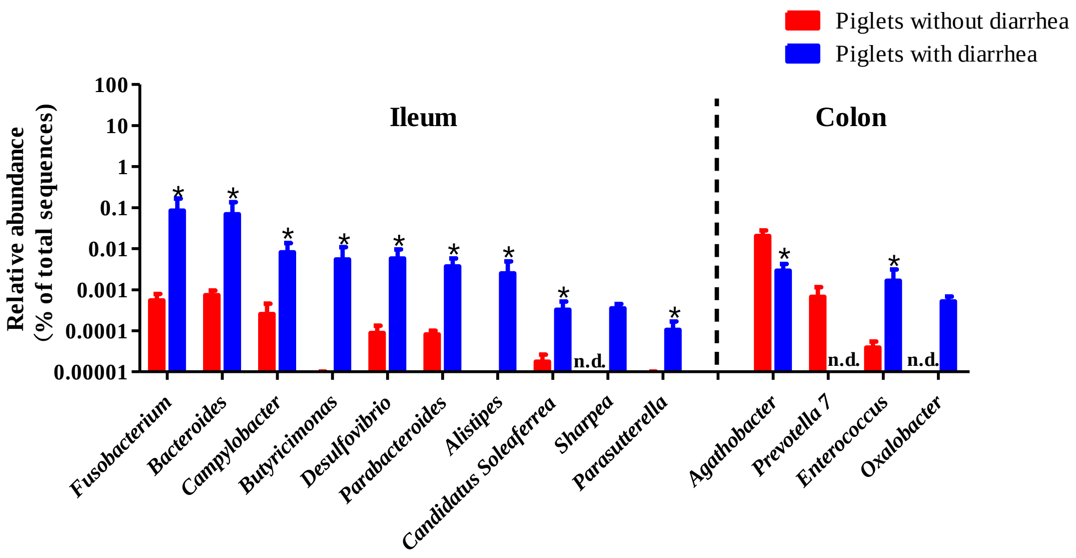


**Figure S10.** Significantly changed genera in the ileal and colonic digesta of piglets with diarrhea (n=3) and without diarrhea (n=7). The values are expressed as the means ± SEM. Asterisks indicate statistical differences between different group. *$P < 0.05$. n.d., no sequence detected.

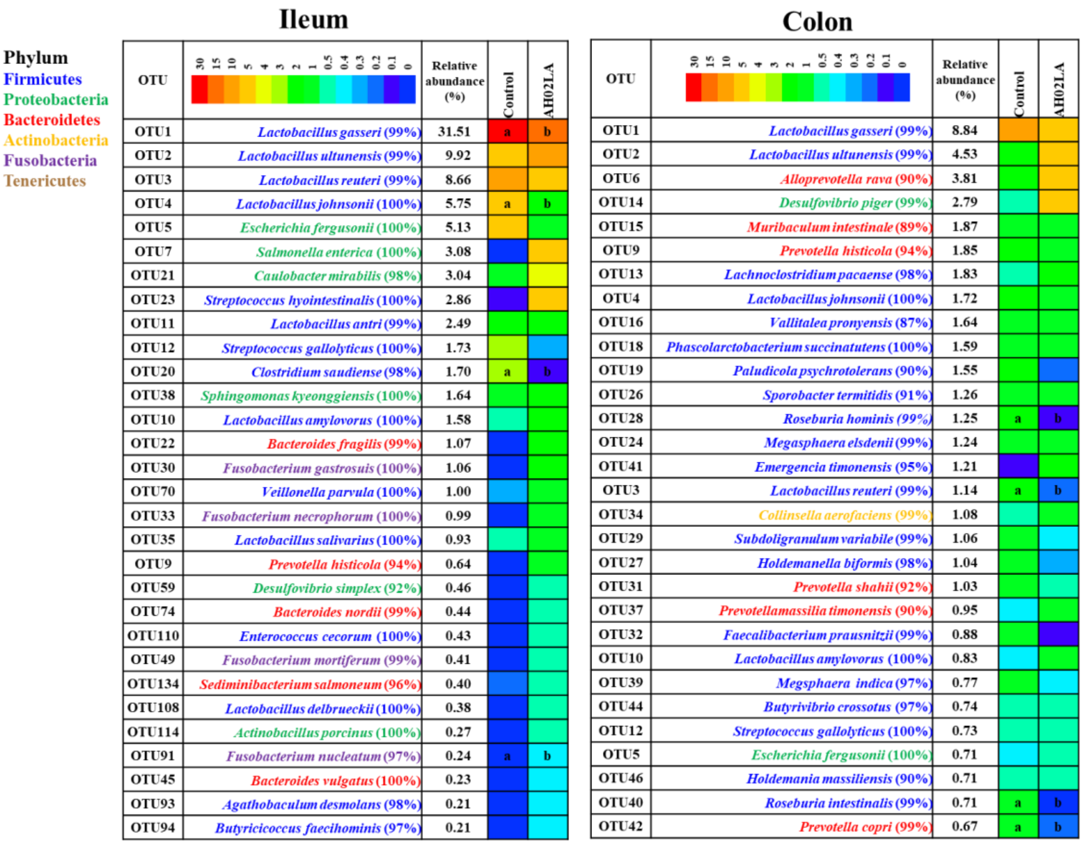


**Figure S11.** Heatmap of dominant OTUs in the ileum and colon of piglets with control and AH02LA groups. a,b means in a row with different superscripts differ. (Mann–Whitney U test and a false discovery rate < 0.05)

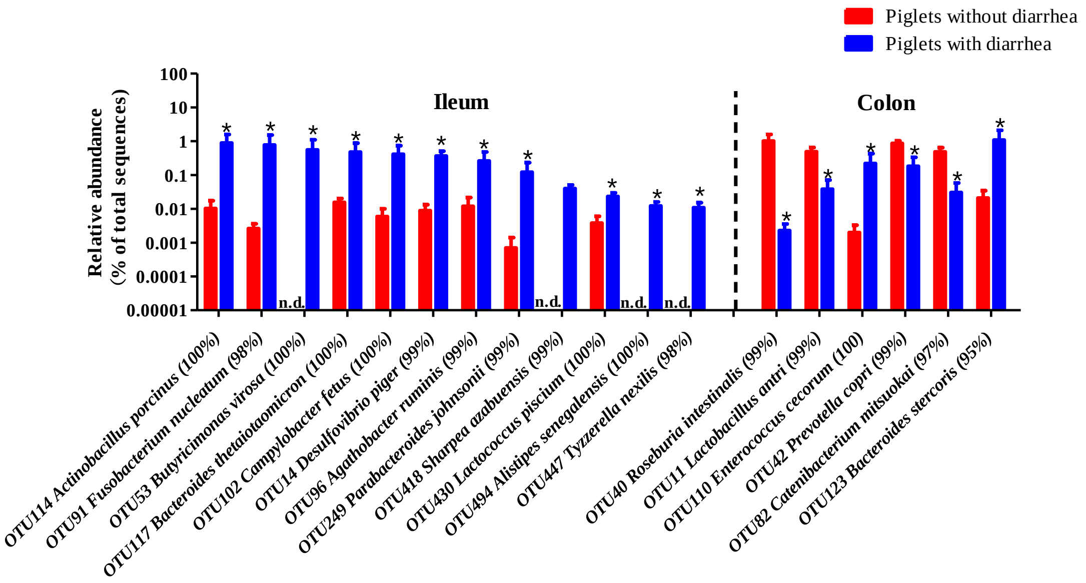


**Figure S12.** Significantly changed bacteria OTUs in the ileal and colonic digesta of piglets with diarrhea (n=3) and without diarrhea (n=7). The values are expressed as the means ± SEM. Asterisks indicate statistical differences between different group. *$P$ < 0.05. n.d., no sequence detected.

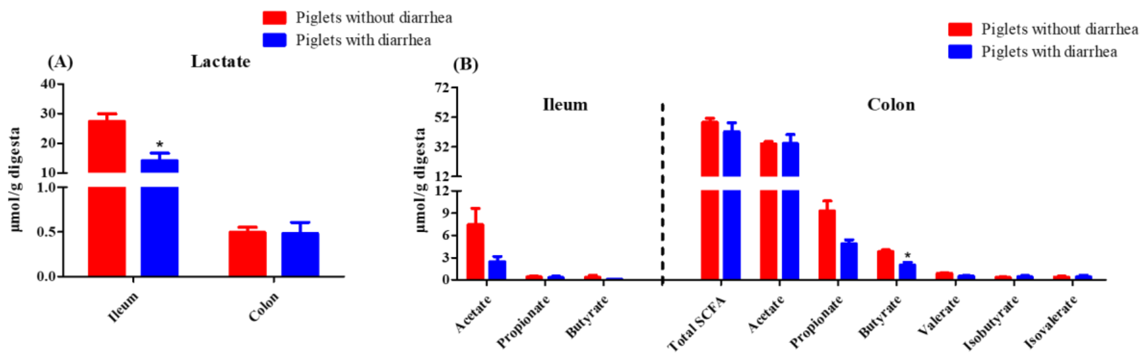


**Figure S13.** The concentrations of lactate (A) and SCFA (B) in the ileal and colonic digesta of piglets with diarrhea (n=3) and without diarrhea (n=7). The values are expressed as the means ± SEM. Asterisks indicate statistical differences between different group. **P* < 0.05. SCFA, short-chain fatty acid.

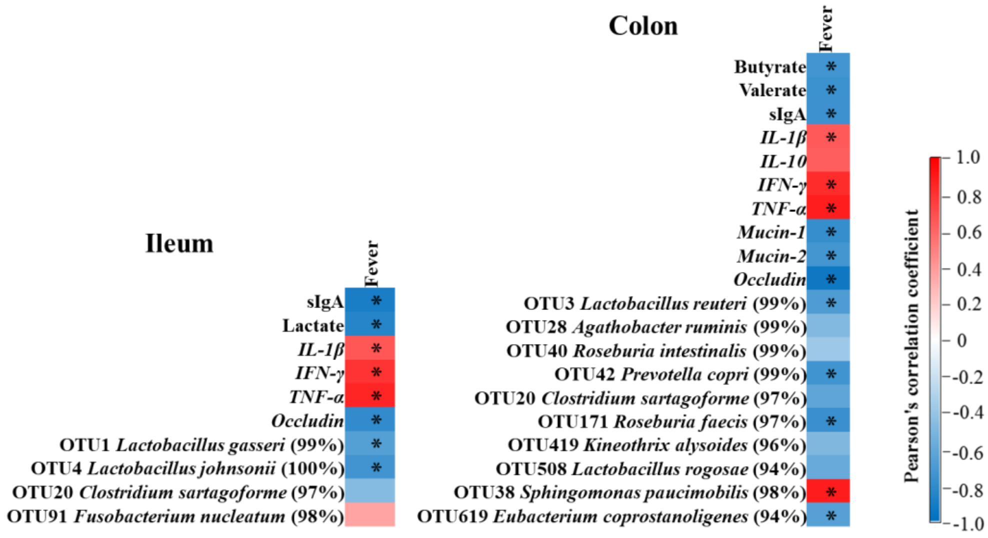


**Figure S14.** Correlation analysis between rectal temperature with immune markers, bacteria or bacterial fermentation products in the ileum and the colon. The red represents a significant positive correlation, and the blue represents a significant negative correlation. **P* < 0.05. *IL*, interleukin; *IFN-γ*, interferon-γ; *TNF-α*, tumor necrosis factor-α; sIgA, secretory immunoglobulin A.

## References


1. Pieper, R.; Krã¶Ger, S.; Richter, J.F.; Wang, J.; Martin, L.; Bindelle, J.; Htoo, J.K.; Von, S.D.; Vahjen, W.; Zentek, J. Fermentable fiber ameliorates fermentable protein-induced changes in microbial ecology, but not the mucosal response, in the colon of piglets. *J. Nutr.* **2012**, *142*, 661-667.
2. Fang, L.; Jiang, X.; Su, Y.; Zhu, W. Long-term intake of raw potato starch decreases back fat thickness and dressing percentage but has no effect on the longissimus muscle quality of growing-finishing pigs. *Livest. Sci.* **2014**, *170*, 116-123.
3. Genlai, L.; Wen, Y.; Honglin, J. Short-chain fatty acids enhance adipocyte differentiation in the stromal vascular fraction of porcine adipose tissue. *J. Nutr.* **2014**, *144*, 1887.
4. Zhou, X.L.; Kong, X.F.; Lian, G.Q.; Blachier, F.; Geng, M.M.; Yin, Y.L. Dietary supplementation with soybean oligosaccharides increases short-chain fatty acids but decreases protein-derived catabolites in the intestinal luminal content of weaned Huanjiang mini-piglets. *Nutr. Res.* **2014**, *34*, 780-788.
5. Villodre Tudela, C.; Boudry, C.; Stumpff, F.; Aschenbach, J.R.; Vahjen, W.; Zentek, J.; Pieper, R. Down-regulation of monocarboxylate transporter 1 (MCT1) gene expression in the colon of piglets is linked to bacterial protein fermentation and pro-inflammatory cytokine-mediated signalling. *Br. J. Nutr.* **2015**, *113*, 610-617.
6. Chang, C.; Zhang, D.H.; Tang, B.; Liu, G.Y.; Hua, T.; Hou, J.B. Development of SYBR-Green Ⅰ real-time quantitative PCR assay for detection of Pseudorabies wild-type virus. *Acta Agriculturae Jiangxi.* **2017**, *29*, 1-4.
